# Association between per- and polyfluoroalkyl substances and risk of hypertension: a systematic review and meta-analysis

**DOI:** 10.3389/fpubh.2023.1173101

**Published:** 2023-08-02

**Authors:** Fang Xiao, Ziwen An, Junli Lv, Xiaoyi Sun, Heming Sun, Yi Liu, Xuehui Liu, Huicai Guo

**Affiliations:** ^1^Department of Toxicology, School of Public Health, Hebei Medical University, Shijiazhuang, China; ^2^Department of Occupational and Environmental Health, School of Public Health, Hebei Medical University, Shijiazhuang, China; ^3^Hebei Key Laboratory of Environment and Human Health, Shijiazhuang, China

**Keywords:** per-and polyfluoroalkyl substances, hypertension, high blood pressure, meta-analysis, environmental pollutants

## Abstract

**Background:**

Existing evidence indicates that exposure to per- and polyfluoroalkyl substances (PFASs) may increase the risk of hypertension, but the findings are inconsistent. Therefore, we aimed to explore the relationship between PFASs and hypertension through this systematic review and meta-analysis.

**Methods:**

We searched PubMed, Embase, and the Web of Science databases for articles published in English that examined the relationship between PFASs and hypertension before 13 August 2022. The random effects model was used to aggregate the evaluation using Stata 15.0 for Windows. We also conducted subgroup analyses by region and hypertension definition. In addition, a sensitivity analysis was carried out to determine the robustness of the findings.

**Results:**

The meta-analysis comprised 15 studies in total with 69,949 individuals. The risk of hypertension was substantially and positively correlated with exposure to perfluorooctane sulfonate (PFOS) (OR = 1.31, 95% CI: 1.14, 1.51), perfluorooctanoic acid (PFOA) (OR = 1.16, 95% CI: 1.07, 1.26), and perfluorohexane sulfonate (PFHxS) (OR = 1.04, 95% CI: 1.00, 1.09). However, perfluorononanoic acid (PFNA) exposure and hypertension were not significantly associated (OR = 1.08, 95% CI: 0.99, 1.17).

**Conclusion:**

We evaluated the link between PFASs exposure and hypertension and discovered that higher levels of PFOS, PFOA, and PFHxS were correlated with an increased risk of hypertension. However, further high-quality population-based and pathophysiological investigations are required to shed light on the possible mechanism and demonstrate causation because of the considerable variability.

**Systematic review registration:**

https://www.crd.york.ac.uk/prospero/ PROSPERO, registration number: CRD 42022358142.

## Graphical Abstract

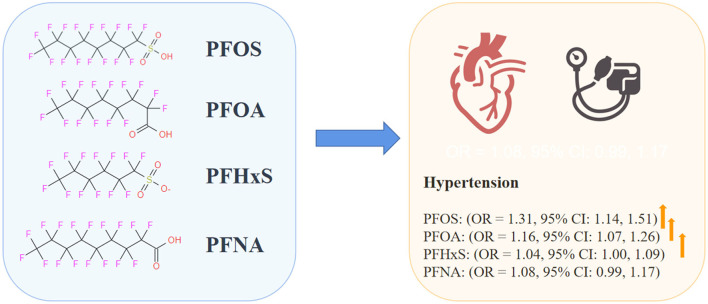

## 1. Introduction

Since the 1940s, per- and polyfluoroalkyl substances (PFASs) have been extensively used because of their surfactant and stability qualities in industrial processes and goods such as aerospace and military, automotive, aviation, textiles, leather, clothing, construction and household goods, electronics, fire protection, food processing, and medical supplies (1–3). Persistent organic pollutants (POPs), including PFASs are now widely found in the environment, animals, plants, and humans worldwide because of their extensive usage (4). Due to the production of fluorocarbon bonds, PFASs have a long half-life and biopersistence in humans. The typical serum half-life of PFASs ranges from 2.3 to 8.5 years (5). Due to their structural similarity to fatty acids, PFASs may interfere with the function of peroxisome proliferator-activated receptors (PPARs) and the signaling pathways that connect them to metabolic processes (6). Meanwhile, toxicological investigations have also indicated that PFASs exposure is connected with oxidative stress and endothelial dysfunction (7). Thus, the cardiovascular system is especially susceptible to the toxicity of PFASs. PFASs exposure is associated with an increased risk of cardiovascular disease (CVD) and peripheral artery disease (PAD) (8, 9), in addition to other CVD risk factors such as thyroid disease (10, 11), high total cholesterol and low-density lipoprotein (LDL) levels (12), a higher body mass index (13), and impaired glucose homeostasis.

According to the data from the World Burden of Disease (GBD), the increasing incidence of hypertension has emerged as a major contributor to global mortality (14–17). Hypertension is also a significant contributor to the development of cardiovascular disease and renal failure (18). Different environmental exposures, including nutrition, alcohol consumption, lifestyle, and environmental contaminants, have been found to have variable impacts on blood pressure (19–21) and have been implicated as essential and changeable risk factors for hypertension (22). Toxicological evidence shows that PFASs may contribute to hypertension by increasing oxidative stress and the generation of reactive oxygen species (23). Several cross-sectional studies have shown that there is a positive correlation between PFASs exposure (24–29) and hypertension, while some researchers have reported no correlation or even a negative correlation (30–38). Conclusions vary depending on the population studied and the specific PFASs, and substantial inconsistencies have been observed between multiple studies on the same type of PFASs. Furthermore, PFASs exposure has occurred worldwide but still varies among countries due to the diversity of potential sources and approaches (39). These results show that more research is needed to gather data and quantify the impact of lingering, alternative, and emergent fluorinated chemicals on the blood pressure health of the population.

A systematic review and meta-analysis were conducted to (1) review the evidence for the effect of PFASs exposure on hypertension in the population and (2) quantitatively assess the relationship between the concentration of specific PFASs in the blood and the risk of hypertension.

## 2. Methods

### 2.1. Data sources and search strategies

The review has been registered in the International Prospective Register of Systematic Reviews (PROSPERO; registration number: CRD 42022358142) (https://www.crd.york.ac.uk/prospero/), which was conducted under the guidance of the Preferred Reporting Items for Systematic Reviews and Meta-Analyses (PRISMA) statement (40). All the relevant studies in the database were searched from its establishment to 13 August 2022 to obtain all relevant articles from the PubMed, Embase, and Web of Science databases. Search keywords include exposure (per- and polyfluoroalkyl substances, PFOS, PFOA, PFNA, and PFHxS) and result (hypertension). A specific search strategy is added to the Supplementary material. Given the complexity and growing number of PFASs homologs, we manually scanned all the references in the collected research to obtain more relevant articles and ensure that all investigations were included.

### 2.2. Selection criteria

We preliminarily screened the titles and abstracts, evaluated the full articles, and independently identified articles that met the criteria. The following epidemiological studies are included: (1) observational study design, such as case–control studies, cohort studies, and cross-sectional studies; (2) at least one type of PFASs exposure (such as PFOS, PFOA, PFNA, and PFHxS) is observed; (3) hypertension results; (4) a risk assessment is provided, including a 95% confidence interval (CI), ORs, RRs, or HRs. Exclusion criteria included studies that (1) are not full-text; (2) have pregnant women as participants; (3) have repetitive data; (4) take the form of laboratory research, non-human animal research, a letter, or a review; and (5) are of low quality.

### 2.3. Data extraction and quality assessment

Lv and An separately extracted data and evaluated the quality of each research project. Disagreements were discussed and resolved amicably. We retrieved the following data from every qualifying study: first author; publication year; research design; population characteristics (distribution of region, age, and gender); sample size; categories of PFASs; definition and diagnostic criteria of hypertension; maximum adjusted ORs, RRs, or HRs, 95% CI (41) corresponding adjustment covariates and so on.

The Newcastle–Ottawa Scale (NOS) was used in order to assess the level of methodological rigor present in case–control studies and cohort studies (42). The study's quality was evaluated based on its selection, comparability, exposure (in case–control studies), or result (in cohort studies). The highest score was 9, and research that scored ≥ 7 was considered high quality. The cross-sectional scale recommended by the Agency for Healthcare Research and Quality (AHRQ) was used to evaluate cross-sectional research (43). The scale consists of 11 items, with a maximum score of 11. The evaluation criteria are as follows: 0–3 = low quality, 4–7 = medium quality, and 8–11 = high quality.

The NTP/OHAT Risk of Bias Rating was also used to assess the quality of the included studies (44). Seven main domains were included: selection bias, confounding bias, attrition/exclusion bias, exposure characteristics, outcome representation, selective reporting bias, and conflict of interest. The criteria for risk of bias assessment are reported in Supplementary Table 3.

The Grading of Recommendations Assessment, Development, and Evaluation (GRADE) guideline was used to assess the confidence in the body of evidence (45), which evaluates eight criteria (risk of bias, indirectness, inconsistency, imprecision, publication bias, large magnitude of effect, dose–response, and confounding effect) to systematically assess the overall confidence in the evidence derived from the meta-analysis. Based on the overall assessment of reviewers, the method assigns the evidence a quality rating of “high,” “moderate,” “low,” or “very low.”

### 2.4. Statistical analysis

The ORs, RRs, and HRs, together with their respective 95% CIs, were derived using the maximum adjusted models in each research study. For these studies in which the categorical PFASs exposure dosage was variable and split into tertiles or quartiles, the fixed effect model combined the data, and the meta-analysis used the final pooled findings (46). The relevant effect values, such as ORs, RRs, or HRs, which may be integrated into the meta-analysis, were included. The effect size (ES) was calculated by ES = ln (OR), and the standard error (SE) of the effect size was calculated as SE = [ln (UC)–ln (LC)]/3.92 (UC and LC represent upper and lower confidence limits, respectively). The % weight represented the size of the information (i.e., sample size, number of events, and confidence interval) and was calculated as weight = 1/(SE^2^) (41). Heterogeneity in the research was tested using the *I*^2^ and *P*-values. A *P*-value of < 0.05 was regarded as heterogeneous. *I*^2^ statistics > 50% showed high heterogeneity, 25–50% moderate heterogeneity, and < 25% low heterogeneity. The fixed effect model was utilized for analysis when there was no significant heterogeneity (*I*^2^ < 50% or *P* > 0.05), otherwise, a random effect model was employed (46).

To determine the cause of the variation, a subgroup analysis was performed, stratified by geographic area and hypertension threshold. To evaluate the impact of missing studies and to identify the source and size of any heterogeneity in the results, sensitivity analyses were conducted by eliminating studies one by one from the analysis. Publication bias was evaluated using funnel plots, and the predicted findings were confirmed using Egger's test (47). The meta-analysis used Stata version 15.0 for Windows.

## 3. Results

### 3.1. Study selection

**Figure 1:**
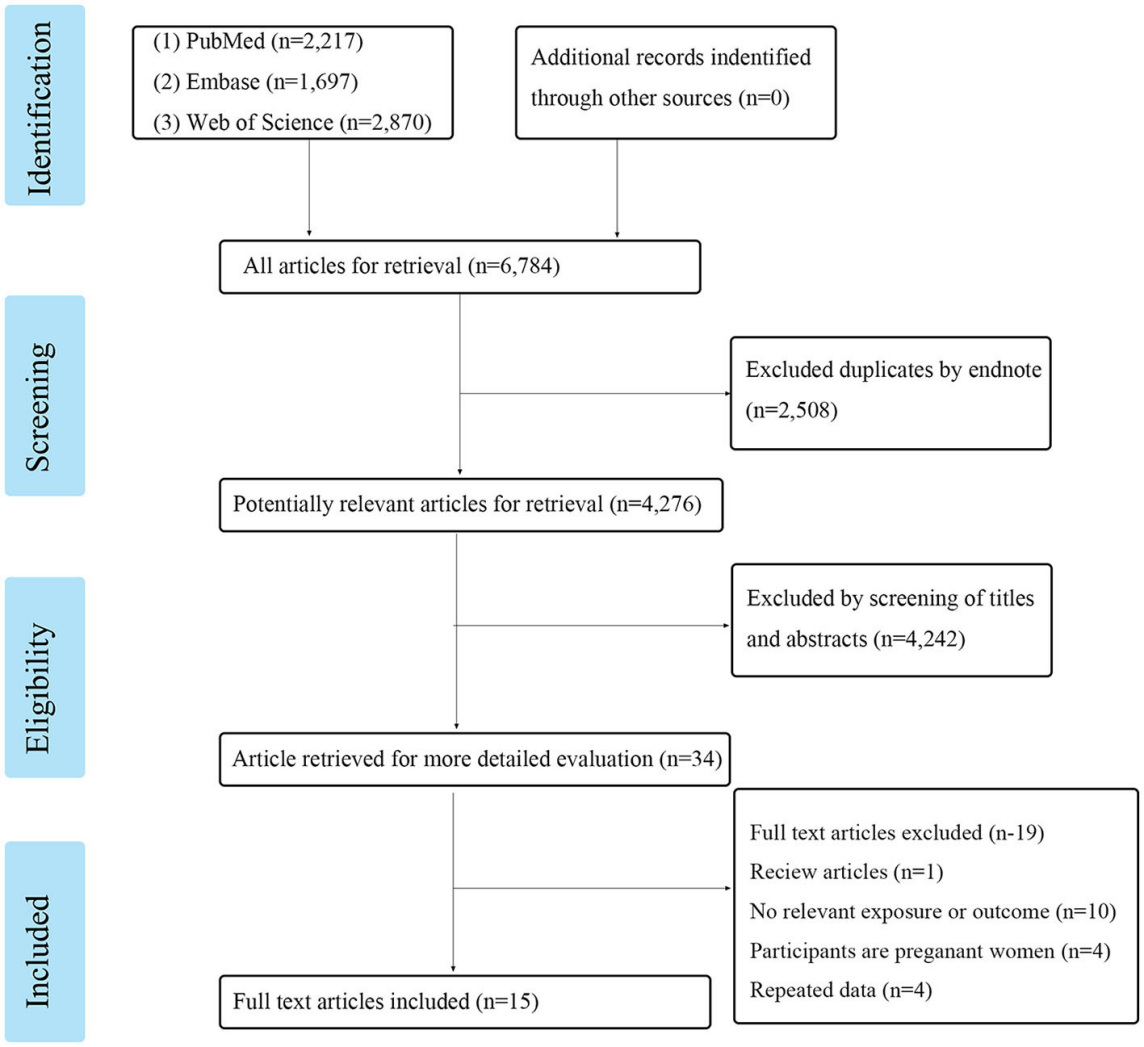
PRISMA flow diagram of study selection.

Following the search strategy, a total of 6,784 articles from these three online resources were reviewed. After removing the duplicates, we were left with 4,276 research articles. After evaluating the titles and abstracts, 34 articles were chosen for further consideration. Researchers discarded 19 articles because they did not meet the inclusion or exclusion criteria. These included one review, 10 studies without an applicable exposure or result, four studies conducted on pregnant women, and four studies that simply replicated previous findings. A total of 15 publications were included in the meta-analysis, as shown in Figure 1. There were a total of 71,059 participants in the studies that quantified PFASs levels in blood samples from 154 to 32,254 individuals. Table 1 shows the detailed article information.

**Table 1:** Characteristics of studies included in the meta-analysis.

**References**	**Year**	**Country**	**Research type**	**Age range or mean age (years)**	**Male subjects (%)**	**Exposure to substances**	**Estimated size (95%CI)**	***N***	**Covariates**
Min et al. (26)	2012	United States	Cross-sectional study	≥20	NR	PFOA	Q2-Q1: 1.21 (0.86, 1.70) Q3-Q1: 1.60 (1.15, 2.22) Q4-Q1: 1.71 (1.23, 2.36)	2,934	Age, sex, ethnicity, education, income, smoking status, alcohol use, obesity status, total saturated fatty acid intake, physical activity, serum PFOS concentrations, total cholesterol, and poor kidney function
Geige et al. (30)	2014	United States	Cross-sectional study	15 ± 0.1	51	PFOS PFOA	Q2-Q1: 0.99 (0.55, 1.78) Q3-Q1: 0.73 (0.36, 1.48) Q4-Q1: 0.77 (0.37, 1.61) Q2-Q1: 0.89 (0.53, 1.49) Q3-Q1: 0.96 (0.53, 1.73) Q4-Q1: 0.69 (0.41, 1.17)	1,655	Age, sex, ethnicity, body mass index, annual household income, physical activity, total cholesterol, and serum cotinine
Winquist and Steenland (24)	2014	United States	Cohort study	≥20	46	PFOA	Q2-Q1: 1.10 (1.02, 1.19) Q3-Q1: 1.10 (1.02, 1.18) Q4-Q1: 1.05 (0.97, 1.12) Q5-Q1: 0.98 (0.91, 1.06)	32,254	Sex, ethnicity, education, smoking status, alcohol use, body mass index, and diabetes
Christensen et al. (34)	2016	United States	Cross-sectional study	≥50	100	PFOS PFOA PFNA PFHxS	0.99 (0.96,1.01) 0.74 (0.52,1.01) 0.74 (0.53,0.98) 1 (0.88,1.13)	154	Age, BMI, work status, and alcohol consumption
Bao et al. (36)	2017	China	Cross-sectional study	55.1 ± 16.4	74.69	PFOS PFOA PFNA PFHxS	1.24 (1.08, 1.44) 1.12 (0.97, 1.30) 1.19 (1.04, 1.36) 0.99 (0.95, 1.03)	1,612	Age, sex, BMI, education, income, exercise, smoking status, alcohol consumption, and family history of hypertension
Chen et al. (38)	2019	Croatia	Cross-sectional study	55 ± 15.8	42.10	PFOS PFOA PFNA PFHxS	1.24 (0.67, 2.31) 0.72 (0.32, 1.63) 0.89 (0.39, 2.04) 1.14 (0.63, 2.05)	1,430	Age, sex, education, socio-economic status, smoking status, diet, physical activity
Donat-Vargasa et al. (32)	2019	Sweden	Case–control study	56 ± 6	54	PFOS PFOA PFNA PFHxS	Q2-Q1: 0.82 (0.48, 1.40) Q3-Q1: 0.73 (0.41, 1.30) Q2-Q1: 0.79 (0.47, 1.33) Q3-Q1: 0.87 (0.50, 1.52) Q2-Q1: 0.96 (0.59, 1.56) Q3-Q1: 0.90 (0.52, 1.57) Q2-Q1: 1.16 (0.58, 2.33) Q3-Q1: 0.54 (0.25, 1.18)	370	Age, sex, education, year of sampling, body mass index, smoking status, alcohol consumption, physical activity, and healthy diet score
Liao et al. (25)	2020	United States	Cross-sectional study	49.9 ± 18	50.60	PFOS PFOA PFNA PFHxS	Q2-Q1: 1.16 (0.99, 1.35) Q3-Q1: 1.14 (0.97, 1.34) Q3-Q1: 1.04 (0.90, 1.21) Q3-Q1: 1.35 (1.16, 1.58) Q2-Q1: 1.18 (1.01, 1.37) Q3-Q1: 1.18 (1.01, 1.38) Q2-Q1: 1.07 (0.92, 1.24) Q3-Q1: 1.19 (1.02, 1.38)	6,967	Age, sex, education level, ethnicity, diabetes mellitus, consumption of at least 12 drinks/year, current smoking status, body mass index, and waist circumference
Pitter et al. (35)	2020	Italy	Cross-sectional study	20-39	48.57	PFOS PFOA PFHxS PFNA	Q2-Q1: 0.99 (0.85, 1.16) Q3-Q1: 1.06 (0.91, 1.24) Q4-Q1: 1.12 (0.95, 1.32) Q2-Q1: 1.00 (0.85, 1.16) Q3-Q1: 1.02 (0.87, 1.20) Q4-Q1: 1.16 (0.99, 1.37) Q2-Q1: 1.01 (0.86, 1.19) Q3-Q1: 1.08 (0.92, 1.27) Q4-Q1: 1.19 (1.00, 1.41) 1.10 (0.96, 1.26)	1,430	Age, sex, education, socioeconomic status, smoking, diet, physical activity
Mi et al. (27)	2020	China	Cross-sectional study	61.98 ± 14.40	54.85	PFOS PFOA	2.52 (1.91, 3.33) 1.72 (1.27, 2.31)	1,238	Age, sex, ethnicity, occupation, education, smoking, alcohol consumption, physical activity, annual household income, and seafood consumption
Lin et al. (37) (1)	2020	United States	Cohort study	NA	34.70	PFOS PFOA PFNA PFHxS	Q2-Q1: 1.09 (0.76, 1.54) Q3-Q1: 1.18 (0.84, 1.66) Q4-Q1: 1.19 (0.85, 1.67) Q2-Q1: 1.15 (0.84, 1.58) Q3-Q1: 1.08 (0.79, 1.48) Q4-Q1: 1.24 (0.91, 1.68) Q2-Q1: 0.78 (0.54, 1.11) Q3-Q1: 1.02 (0.78, 1.34) Q4-Q1: 1.00 (0.76, 1.32) Q2-Q1: 1.42 (0.94, 2.17) Q3-Q1: 1.49 (0.98, 2.25) Q4-Q1: 1.59 (1.05, 2.41)	957	Age, sex, ethnicity, treatment assignment, education, income, marital status, alcohol consumption, smoking, and DASH diet score
Lin et al. (37) (2)	2020	United States	Cohort study	NA	34.70	PFOS PFOA PFNA PFHxS	Q2-Q1: 1.65 (0.94, 2.88) Q3-Q1: 1.58 (0.91, 2.74) Q4-Q1: 1.45 (0.83, 2.52) Q2-Q1: 1.15 (0.78, 1.68) Q3-Q1: 0.96 (0.65, 1.41) Q4-Q1: 0.95 (0.65, 1.38) Q2-Q1: 1.07 (0.78, 1.48) Q3-Q1: 0.96 (0.70, 1.31) Q4-Q1: 0.93 (0.68, 1.28) Q2-Q1: 1.08 (0.83, 1.42) Q3-Q1: 1.09 (0.83, 1.41) Q4-Q1: 0.84 (0.60, 1.18)	956	Age, sex, ethnicity, treatment assignment, education, income, marital status, alcohol drinking, smoking, and DASH score
Averina et al. (31)	2021	Norway	Cross-sectional study	16.30 ± 1.24	52.66	PFOS PFOA PFHxS	Q2-Q1: 1.40 (0.78, 2.51) Q3-Q1: 1.01 (0.56, 1.80) Q4-Q1: 1.86 (1.08, 3.19) Q2-Q1: 1.28 (0.74, 2.22) Q3-Q1: 1.45 (0.85, 2.49) Q4-Q1: 2.08 (1.17, 3.69) Q2-Q1: 1.63 (0.90, 2.94) Q3-Q1: 1.25 (0.69, 2.28) Q4-Q1: 2.06 (1.16, 3.65)	940	Sex, age, BMI, and physical activity outside of school
Zare Jeddi et al. (33)	2021	Italy	Cross-sectional study	30 ± 5.8	48.61	PFOS PFOA PFNA PFHxS	1.10 (1.03, 1.17) 1.05 (1.01, 1.08) 1.07 (1.03, 1.12) 1.10 (0.99, 1.21)	15,876	Age, gender, time between study entry and blood sampling center where BP was measured, education, number of deliveries, physical activity, country of birth, diet, alcohol intake, smoking status, HDL-C, BMI ≥ 25, diabetes
Yu et al. (28)	2021	China	Cross-sectional study	61.8 ± 14.4	55.30	PFOS	Q2-Q1: 4.19 (2.89, 6.08) Q3-Q1: 3.29 (2.27, 4.75) Q4-Q1: 5.53 (3.72, 8.23)	1,228	Age, sex, annual income, smoking, alcohol consumption, physical activity, and seafood consumption
Ding et al. (29)	2022	United States	Cohort study	49.2	0	PFOS PFOA PFNA PFHxS	Q2-Q1: 1.11 (0.93, 1.33) Q3-Q1: 1.42 (1.19, 1.68) Q2-Q1: 1.37 (1.15, 1.63) Q3-Q1: 1.47 (1.24, 1.75) Q2-Q1: 0.93 (0.79, 1.09) Q3-Q1: 1.00 (0.83, 1.19) Q2-Q1: 0.84 (0.71, 1.00) Q3-Q1: 1.06 (0.89, 1.25)	1,058	Ethnicity, study site, education, financial strain, smoking status, environmental tobacco smoke, alcohol consumption, total caloric intake, and menopausal status

BMI, body mass index; HDL-C, high-density lipoprotein cholesterol; DASH, dietary approaches to hypertension; PFOS, perfluorooctane sulfonate; PFOA, perfluorooctanoic acid; PFNA, perfluorononanoic acid; PFHxS, perfluorohexane sulfonate; Q1, first or lowest quartile; Q2, second quartile; Q3, third quartile; Q4, fourth quartile; Q5, fifth quartile; DASH score, Dietary Approaches to Stop Hypertension score; NA, not applicable; NR, not reported.

### 3.2. Definition of hypertension

The term “hypertension” alone has a wide range of interpretations. The majority of studies (10 of 15 included studies) use a blood pressure reading of 140/90 mm Hg as the diagnostic threshold for hypertension (24–27, 29, 30, 32, 35–37), which is in line with the guidelines of the American Heart Association and the Joint National Committee (Seventh Report of the Joint National Committee on Prevention, Detection, Evaluation, and Treatment of High Blood Pressure). Four studies use a blood pressure reading of 130/85 mm Hg as the cutoff for hypertension (28, 33, 38), while another (31) uses a reading of 130/80 mm Hg since its subjects are adolescents. An individual study did not definitively establish the cutoff for hypertension, with the condition being defined as one that requires a medical professional's diagnosis (34).

### 3.3. Assessment of quality

The quality of the 15 studies that were suitable for inclusion was analyzed. The results showed that 11 of the cross-sectional studies were of high- or medium-quality (25–31, 33–36, 38), three of the cohort studies scored 7 (24, 29, 37), and one case–control study scored 8 (32), indicating that none of these studies was of poor quality. Supplementary Tables 1, 2 include further information.

The results of the risk of bias assessment are shown in Table 2. The risk of bias regarding attrition/exclusion, confounding, selection (exposure), and conflict was rated as “probably low” in all the included studies. Of the 15 studies, five were rated as “definitely low risk of bias”, seven were rated as “probably low”, and two were rated as “definitely high risk of bias” due to the use of self-reported cases. Selection bias was rated as “probably low” in all but one study. Overall, 13 studies and two studies were grouped as tier 1 and tier 2, respectively.

**Table 2:** Summary of risk of bias domains for individual studies examining associations between PFAS and hypertension.

**RESPONSE LEVEL**		**Min et al. (26)**	**Geige et al. (30)**	**Winquist and Steenland (24)**	**Christensen et al. (34)**	**Bao et al. (36)**	**Chen et al. (38)**	**Donat-Vargasa et al. (32)**	**Liao et al. (25)**	**Pitter et al. (35)**	**Mi et al. (27)**	**Lin et al. (37)**	**Averina et al. (31)**	**Zare Jeddi et al. (33)**	**Yu et al. (28)**	**Ding et al. (29)**
++	Definitely low risk of bias															
+	Probably low risk of bias															
-	Probably high risk of bias															
–	Definitely high risk of bias															
**BIAS MAIN**																
**CONFOUNDING BIAS**. [Key domain] Did the study design or analysis account for important confounding and modifying variables?		+	+	+	+	+	+	+	+	+	+	+	+	+	+	+
**ATTRITION/EXCLUSION BIAS** Were outcome data incomplete due to attrition or exclusion from the analysis?		+	+	+	+	+	+	+	+	+	+	+	+	+	+	+
**DETECTION BIAS** Can we trust the exposure characterization? [Key domain]		+	+	+	+	+	+	+	+	+	+	+	+	+	+	+
Can we be confident in the outcome assessment? [Key domain]		+	++	-	-	++	+	++	++	+	++	+	+	+	++	+
**SELECTIVE REPORTING BIAS** Were all measured outcomes reported?		+	+	+	+	+	+	+	+	+	+	+	+	+	+	+
**SELECTION BIAS** Did the selection of study participants result in appropriate comparison groups?		+	+	+	-	+	+	+	+	+	+	+	+	+	+	+
**CONFLICT OF INTEREST**		+	+	+	+	+	+	+	+	+	+	+	+	+	+	+
**SUMMARY TIERED CLASSIFICATION**		T1	T1	T2	T2	T1	T1	T1	T1	T1	T1	T1	T1	T1	T1	T1

“T1” is Tier 1: the study must be rated as “definitely low” or “probably low” risk of bias for key criteria AND “definitely low” or “probably low” risk of bias for most other applicable criteria. “T2” is Tier 2: the study does not meet the criteria for tiers 1 or 2. Tier 3: the study must be rated as “definitely high” or “probably high” risk of bias for key criteria AND “definitely high” or “probably high” risk of bias for most other applicable criteria. Tier 3: the study must be rated as “definitely high” or “probably high” risk of bias for key criteria AND “definitely high” or “probably high” risk of bias for most other applicable criteria.

Based on cross-sectional and case–control studies, the overall strength of evidence for the association between PFASs and hypertension was “limited”, and the direction of effect was inconsistent across most studies. However, for PFASs combined with hypertension events, we rated the overall strength of evidence as “moderate”. The majority of PFASs–hypertension combinations assessed exhibited consistent statistically significant positive evidence of an association, and all studies included in the meta-analysis were “moderate.” These results provide some epidemiologic proof that PFASs may increase the risk of hypertension.

### 3.4. Meta-analysis

**Figure 2:**
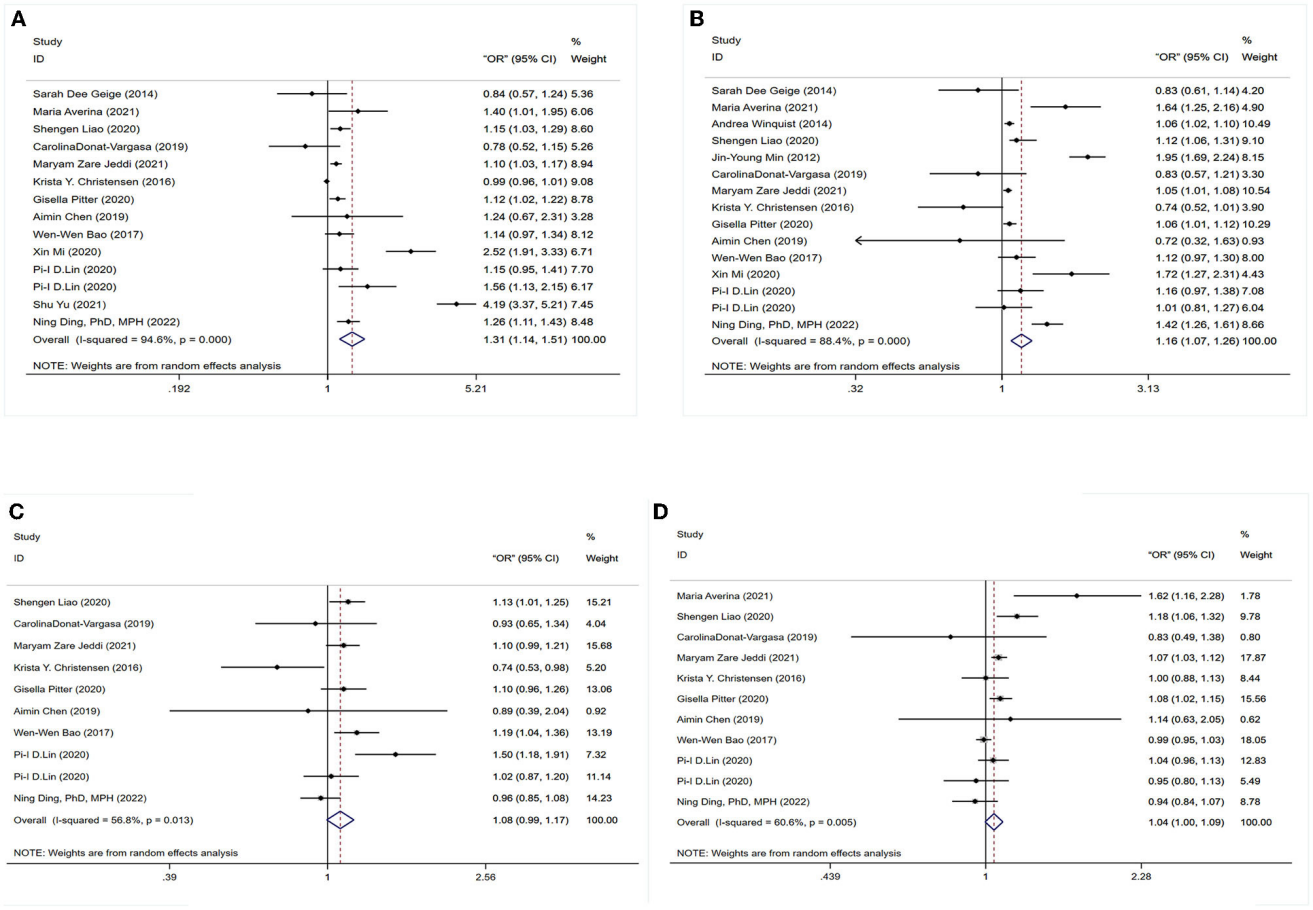
Forest plot of the association between exposure to PFAS with hypertension. **(A)** PFOS; **(B)** PFOA; **(C)** PFNA; **(D)** PFHxS.

To determine whether PFASs exposure was associated with hypertension, 15 studies were analyzed, as shown in Figure 2. This included 15 outcomes for PFOA exposure, 14 outcomes for PFOS exposure, 11 outcomes for PFHxS exposure, and 10 outcomes for PFNA exposure.

#### 3.4.1. Association between PFOS exposure and hypertension

The association between PFOS exposure and hypertension was investigated in 13 studies (10 cross-sectional, one case–control, and two cohort studies). A combined OR estimate of 1.31 (95% CI: 1.14, 1.51) was calculated. We employed a random effects model to examine the connection between PFOS exposure and hypertension due to the significant heterogeneity of the studies (*I*^2^ = 94.6%, *P* < 0.05), as shown in Figure 2A.

#### 3.4.2. Association between PFOA exposure and hypertension

In total, 11 cross-sectional studies, one case–control study, and three cohort studies were retrieved to examine the correlation between PFOA exposure and hypertension. The overall findings revealed that being exposed to PFOA increased the risk of hypertension (OR = 1.16, 95% CI: 1.07, 1.26). A random effects model was used due to the significant heterogeneity of the included studies (*I*^2^ = 88.4%, *P* < 0.05), as shown in Figure 2B.

#### 3.4.3. Association between PFNA exposure and hypertension

No statistically significant association between PFNA exposure and hypertension was found in six cross-sectional studies, one case–control study, and two cohort studies. As a whole, we found a merged evaluation of 1.08 (95% CI: 0.99, 1.17). In addition, a random effects model was adopted since the studies had high heterogeneity (*I*^2^ = 6.8%, *P* < 0.05), as shown in Figure 2C.

#### 3.4.4. Association between PFHxS exposure and hypertension

The association between PFHxS exposure and hypertension risk was examined in seven cross-sectional investigations, one case–control study, and two cohort studies. A positive relationship (OR = 1.04, 95% CI: 1.00, 1.09) was found between PFHxS exposure and the risk of hypertension with a random effect model because of high heterogeneity (*I*^2^ = 60.6%, *P* < 0.05), as shown in Figure 2D.

### 3.5. Subgroup analysis

Exposure to PFOS, PFOA, and PFHxS was observed to have a positive and statistically significant connection with hypertension, but exposure to PFNA did not. We conducted a further subgroup analysis based on geography and hypertension thresholds to delve deeper into the correlation studies. Stratified by region, the pooled evaluated OR of PFOA and hypertension was 1.13 (95% CI: 1.03, 1.24) for Non-American region and 1.15 (95% CI: 0.97, 1.35) for American region. Then, the pooled estimate OR of PFNA and hypertension was 1.11 (95% CI: 1.04, 1.19) for non-America and 1.05 (95% CI: 0.90,1.23) for America. In addition, a subgroup analysis by hypertension threshold revealed a positive association between PFOS and PFOA exposure and the development of hypertension (OR = 1.19, 95% CI: 1.05, 1.28; OR = 1.15, 95% CI: 1.03, 1.28) for 140/90 mmHg, but no statistically significant association (OR = 1.58, 95% CI: 0.90, 2.78; OR = 1.28, 95% CI: 0.83, 1.98) for non-140/90 mmHg. All results are shown in Table 3.

**Table 3:** Subgroup analysis of PFASs (PFOA, PFOS, PFNA, and PFHxS) exposure and risk of hypertension.

**Subgroup**	**Regions**		**Threshold for hypertension**	

	**American**	**Non-Amercian**	**140/90 mmHg**	**Non-140/90 mmHg**
**PFOS**				
Studies (*N*)	7	7	11	3
Pooled ORs	**1.28**	**1.24**	**1.19**	1.58
(95% CI)	**(1.05, 1.55)**	**(1.06, 1.46)**	**(1.05, 1.34)**	(0.90, 2.78)
Heterogeneity (*I^2^, P*)	*I*^2^ *=* 93.0%, *P* < 0.05	*I*^2^ *=* 84.1%, *P* < 0.05	*I*^2^ *=* 87.2%, *P* < 0.05	*I*^2^ *=* 95.6%, *P* < 0.05
**PFOA**				
Studies (*N*)	8	7	13	2
Pooled ORs	1.15	**1.13**	**1.15**	1.28
(95% CI)	(0.97, 1.35)	**(1.03, 1.24)**	**(1.03, 1.28)**	(0.83, 1.98)
Heterogeneity (*I*^2^, *P*)	*I*^2^ *=* 92.4%, *P* < 0.05	*I*^2^ *=* 74.1%, *P* < 0.05	*I*^2^ *=* 88.8%, *P* < 0.05	*I*^2^ *=* 90.1%, *P* < 0.05
**PFNA**				
Studies (*N*)	5	5	9	1
Pooled ORs	1.05	**1.11**	1.17	1.10
(95% CI)	(0.90, 1.23)	**(1.04, 1.19)**	(0.97, 1.18)	(0.99,1.21)
Heterogeneity (*I*^2^, *P*)	*I*^2^ *=* 77.1%, *P* < 0.05	*I*^2^ < 25.0%, *P* > 0.05	*I*^2^ *=* 61.4%, *P* < 0.05	–, –
**PFHxS**				
Studies (*N*)	5	6	9	2
Pooled ORs	1.03	1.06	1.03	1.27
(95% CI)	(0.95, 1.11)	(0.99, 1.13)	(0.98, 1.08)	(0.85, 1.90)
Heterogeneity (*I*^2^, *P*)	*I*^2^ *=* 55.1%, *P* < 0.05	*I*^2^ *=* 69.6%, *P* < 0.05	*I*^2^ *=* 48.6%, *P* < 0.05	*I*^2^ *=* 82.5%, *P* < 0.05

Bold indicates statistical significance. PFOS, perfluorooctane sulfonate; PFOA, perfluorooctanoic acid; PFNA, perfluorononanoic acid; PFHxS, perfluorohexane sulfonate.

### 3.6. Sensitivity analysis and publication bias

**Figure 3:**
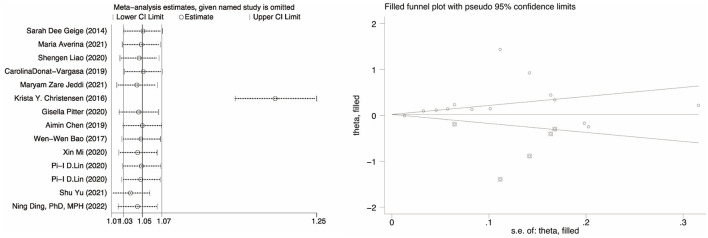
Sensitivity analysis of studies that evaluate the ORs of PFOS.

The correlation between PFOA, PFNA, and hypertension was investigated, and the results showed no substantial publication bias (*P* = 0.26 for PFOA, *P* = 0.56 for PFNA, *P* = 0.67 for PFHxS). However, publication bias was present in the meta-analysis evaluating the association between PFOS and hypertension (P = 0.028). A sensitivity analysis was conducted by eliminating specific articles one by one to assess the stability of the findings and establish that any research did not influence them. The results for PFOA, PFNA, and PFHxS are consistent to some degree. The odds ratio (OR) between PFOS exposure and hypertension increased but remained positively associated (OR = 1.20, 95% CI: 1.16, 1.25), except for the research by Christensen et al. (34). As shown in Figure 3, however, when the trimming and filling approach was used, the result was reversed (OR = 1.04, 95% CI: 0.89, 1.20), indicating that the robustness of the meta-analysis between PFOS and hypertension is poor, and the source of the disagreement must be explained.

## 4. Discussion

In this systematic review and meta-analysis, we first summarize all the current evidence on the risk of PFASs exposure for hypertension. The same type of research on PFASs was collected for analysis in order to summarize their relevance in this study. According to our results, there was a significant positive association between exposure to PFOS, PFOA, and PFHxS and an increased risk of hypertension in the population, but no association between PFNA and hypertension. Our study is the first meta-analysis to investigate the association between PFASs exposure and the risk of hypertension in a population. It has implications for reducing the risk of hypertension in populations living in areas contaminated with PFASs.

The results revealed a substantial positive relationship between PFOA (OR = 1.31, 95% CI: 1.14, 1.51) and PFOS (OR = 1.16, 95% CI: 1.07, 1.26) exposure and the risk of hypertension. Our findings are consistent with the nine previously published studies (25, 27–29, 31, 33, 35–37) that found a positive connection between PFOS or PFOA exposure and hypertension. A negative link between PFOS and hypertension was found in three studies (30, 32, 34), although the results were not statistically significant. Uncontrolled variables, such as diet, comorbidities, physiological features of distinct subpopulations, and family history of hypertension, may be to blame for this discrepancy. For PFHxS, we found a relationship between exposure and hypertension (OR = 1.04, 95% CI: 1.00, 1.09). Our findings are consistent with those of previous research showing that prolonged exposure to high levels of PFHxS increases blood pressure in the general population (25, 27, 31, 35). Although not statistically significant, some studies have shown an inverse correlation between PFHxS and hypertension (29, 33, 34, 36, 37). It is essential to consider the possibility that this discrepancy is due to random chance or confounding factors in the study design or treatment pharmacokinetics. In light of this discrepancy, more studies are needed to establish a causal relationship between PFHxS and hypertension. Contrary to expectations, we found no evidence that PFNA exposure increased the risk of developing hypertension (OR = 1.08, 95% CI: 0.99, 1.17). Studies by Pitter et al. (35) (OR = 1.1, 95% CI: 0.96, 1.26), Lin et al. (37) (OR = 1.00, 95% CI: 0.76, 1.32), Donat-Vargas et al. (32) (OR = 0.9, 95% CI: 0.52, 1.57), and Ding et al. (29) (OR = 1.00, 95% CI: 0.83, 1.19) were consistent with our findings. Three other studies reported positive associations between hypertension and PFNA: one by Liao et al. (25) (OR = 1.18, 95% CI: 1.01, 1.38), one by Zare Jeddi et al. (33) (OR = 1.07, 95% CI: 1.03, 1.12), and one by Bao et al. (36) (OR = 1.19, 95% CI: 1.04, 1.36). However, one study using the NHANCE database found that PFNA exposure was linked to a reduced incidence of hypertension (34) (OR = 0.74, 95% CI: 0.53, 0.98). This finding was not consistent with ours. These discrepancies may be related to the failure to consider other confounding factors that may be strongly associated with hypertension, such as ethnicity, diet, family history, exercise habits, and the local prevalence of hypertension. In addition, this discrepancy highlights the need for further investigation of the effects of PFNA exposure on hypertension.

The association between hypertension and any PFASs (PFOS, PFOA, PFNA, and PFHxS) (OR = 1.26, 95% CI: 1.12, 1.41) was reported in a cohort-based study based on electronic health records (48). Higher blood PFASs concentrations were also related to an increased risk of hypertension (HR = 1.71, 95% CI: 1.15, 2.54) (29), according to a recently published cohort study with a mean follow-up of 12.4 years. At the same time, a cross-sectional study of adolescents in northern Norway showed that total PFASs were positively associated with hypertension, with OR=2.24 95% CI: 1.10, 4.54 (31). Moreover, a study based on the Study of Women's Health Across the Nation reported that in a mixed model, there were positive associations between n-PFOS (β = 0.051), Sm-PFOS (β = 0.115), n-PFOA (β = 0.032), PFNA (β = 0.086), and PFHxS (β = −0.074) (29). This is not entirely consistent with the results of our study. The choice of the statistical model, the definition of hypertension, the degree of exposure, and the susceptibility of different populations may have contributed to this difference (49, 50).

It was shown that in the further stratified analysis, grouping according to the hypertension threshold, PFASs exposure, and hypertension were not significantly correlated with the non-140/90 group, which may be due to the lack of studies in the non-140/90 group (PFOS, *N* = 3; PFOA, *N* = 2; PFNA, *N* = 1; PFHxS, *N* = 2). Studies were categorized by country in order to identify regional variations in the association between PFASs exposure and hypertension. Notably, PFOA, PFNA, and PFHxS were not associated with hypertension in the United States. Regional differences in lifestyle, socioeconomic level, local diets, the local incidence of hypertension, average blood pressure, and ethnic adaptability to PFASs exposure may obscure the association between PFASs exposure and hypertension.

While there are various potential processes linking community exposure to PFASs to an increased risk of hypertension, the mechanism of the relationship between PFASs and blood pressure is still unclear. PFASs have been linked to increased oxidative stress in the liver and endothelial cells (23, 51, 52). Inadequate production of nitric oxide and increased production of superoxide, both byproducts of oxidative stress, may contribute to an increase in blood pressure in the process of attenuating vasodilation. Consequently, PFAS-induced oxidative stress may increase the need for homocysteine methyl donors, which in turn may reduce the effectiveness of the body's natural ability to dilate blood vessels (51, 52). The presence of PFASs may also have a secondary effect on blood pressure, according to another theory. Numerous human (53, 54) and animal studies have revealed that reduced nephron endowment and glucocorticoid excess contribute to hypertension. In a recent animal model of hypertension, prenatal exposure to PFASs was shown to reduce nephron endowment and increase renal glucocorticoid receptor (GR) gene expression in the offspring of mothers who were exposed to the investigated chemicals during pregnancy (55). Upregulation of GRs augments the action of glucocorticoids, and the sclerotic and stress-induced natriuretic cycle initiated by a reduction in nephrons may contribute to hypertension. These mechanisms may cooperate with angiotensin II to boost proximal tubular sodium reabsorption (56). As a result, elevated serum PFASs levels may contribute to an indirect increase in blood pressure, especially in the presence of elevated glucocorticoids and diminished nephrons. In conclusion, PFASs exposure has been associated with a possible increase in the incidence of hypertension. Nevertheless, its mechanism in the human body remains unclear and needs further research.

The research has several strengths. First, the number of hypertension patients in our study is greater than that in smaller studies. With such large samples, we could thoroughly explore the link between PFASs and hypertension risk and conduct a nuanced subgroup analysis. Second, to more thoroughly evaluate the association between exposure to hypertension and various PFASs, we used a fixed effects model to pool data from studies in which the PFASs exposure dosage was categorically variable, such as split into tertiles or quartiles. The findings of this meta-analysis can be trusted since none of the 15 studies used to compile it were of poor quality.

Despite these benefits, there are several caveats to our research. First, many studies looked at “residual PFASs” (PFOS and PFOA) and found that the possible relationship could be completely recognized. However, there is a lack of research on “surrogate PFASs”, or polyfluoroalkyl compounds such as F-53B and OBS, which results in a high degree of variability or a hampered capacity to discover possible correlations. Therefore, the lack of association we observed calls for a thorough explanation and further research. At the same time, PFASs exposure occurs worldwide but differs from country to country due to the wide variety of potential sources and routes of exposure. In addition, because each study's population, models, statistical techniques, and adjustments for numerous confounding variables are unique, each study's conclusions may differ. Finally, a recent study highlighted the importance of PFASs isomers and enantiomers (57). Due to their structural differences, different PFASs isomers and enantiomers may have different harmful health consequences. However, due to the need for data, we instead focused on the general direction of previous studies in this area. As the volume of research continues to grow, we will be able to better categorize our results using more precise diagnostic methods. Despite these obstacles, a meta-analysis can answer numerous questions and shed light on the causes of variability in study findings, pointing the way to new avenues of inquiry.

## 5. Conclusion

Our understanding of the relationship between PFASs exposure and hypertension has been strengthened by this meta-analysis, which demonstrates a positive association between PFOS, PFOA, and PFHxS exposure and hypertension but no relationship between PFNA and hypertension. In order to manage hypertension and further lower the prevalence of cardiovascular disease and stroke, people should seriously consider reducing environmental PFASs pollution and PFASs exposure. To further understand these mechanisms, further research should be encouraged.

## Data availability statement

Publicly available datasets were analyzed in this study. This data can be found here: Pubmed, Embase, and Web Of Science.

## Author contributions

FX and XS: integrity of the data, the accuracy of the data analysis, and study concept and design. FX, ZA, and JL: data extraction and analysis. FX: drafting of the manuscript. ZA and JL: study supervision. HG, XL, ZA, JL, XS, HS, and YL: critical revision. All authors reviewed and revised the manuscript, approved the final version for publication, and accepted responsibility for all aspects of the manuscript.
