# Supplementary material for: Association between per- and polyfluoroalkyl substances and risk of hypertension: a systematic review and meta-analysis
Source: Front Public Health. 2023 Aug 2;11:1173101. doi: 10.3389/fpubh.2023.1173101 (PMC10466234; doi:10.3389/fpubh.2023.1173101)
Supplement: Supplementary file 1 [file Table_1.DOCX]

**Supplementary Material**

1. Research strategy

**Pubmed retrieval formula:** ((Perfluorinated) OR (Alkanesulfonic Acids) OR (Fluorine) OR (Fluorine Compounds) OR (Halothane) OR (perfluorooctane sulfonate) OR (perfluorooctanoate) OR (polyfluoroalkyl compounds) OR (Polyfluoroalkyl chemicals) OR (Perfluorinated chemicals) OR (Perfluorooctanoic acid) OR (perfluorooctane sulfonic acid) OR (perfluorinated acid) OR (fluorocarbons) OR (Perfluorinated alkyl substances) OR (Perfluorohexane sulfonate) OR (perfluoroalkyl acids) OR (fluorinated organic compounds)) AND ((hypertension) OR (Blood Pressure, High) OR (Blood Pressures, High) OR (High Blood Pressure) OR (High Blood Pressures))

**web of science** **retrieval formula**：((((((((((((((((((TS=(Perfluorinated)) OR TS=(Alkanesulfonic Acids)) OR TS=(Fluorine)) OR TS=(Fluorine Compounds)) OR TS=(Halothane)) OR TS=(perfluorooctane sulfonate)) OR TS=(perfluorooctanoate)) OR TS=(polyfluoroalkyl compounds)) OR TS=(Polyfluoroalkyl chemicals)) OR TS=(Perfluorinated chemicals)) OR TS=(Perfluorooctanoic acid)) OR TS=(perfluorooctane sulfonic acid)) OR TS=(perfluorinated acid)) OR TS=(fluorocarbons)) OR TS=(Perfluorinated alkyl substances)) OR TS=(Perfluorohexane sulfonate)) OR TS=(perfluoroalkyl acids)) OR TS=(fluorinated organic compounds)) AND (((((TS=(hypertension)) OR TS=(Blood Pressure, High)) OR TS=(Blood Pressures, High)) OR TS=(High Blood Pressure)) OR TS=(High Blood Pressures))

**EMBASE retrieval formula**： 'hypertension'/exp OR 'hypertension' AND 'perfluoro compound'/exp OR 'perfluoro compound' OR 'alkanesulfonic acid'/exp OR 'alkanesulfonic acid' OR 'fluorine'/exp OR 'fluorine' OR 'halothane'/exp OR 'halothane' OR 'perfluorooctanesulfonic acid'/exp OR 'perfluorooctanesulfonic acid' OR 'polyfluoroalkyl compounds' OR 'perfluorooctanoic acid'/exp OR 'perfluorooctanoic acid' OR 'perfluoro compound'/exp OR 'perfluoro compound' OR 'fluorocarbon'/exp OR 'fluorocarbon' OR 'perfluorinated alkyl substance'/exp OR 'perfluorinated alkyl substance' OR 'perfluorohexanesulfonic acid'/exp OR 'perfluorohexanesulfonic acid'

**Table S1.** Newcastle-Ottawa quality assessment scale

| **First Author (year)** | **Quality Indication from Newcastle-Ottawa Scale** | | | | | | | | **Scores** |
| --- | --- | --- | --- | --- | --- | --- | --- | --- | --- |
|  | 1 | 2 | 3 | 4 | 5 | 6 | 7 | 8 |  |
| Carolina Donat-Vargasa (2019) (27) | ✱ | ✱ | ✱ | ✱ | ✱ | ✱ | ✱ | ✱ | 8 |
| Pi-I D. Lin (2020) (33) | - | ✱ | ✱ | ✱ | ✱ | ✱ | ✱ | - | 7 |
| Andrea Winquist (2014) (24) | - | ✱ | ✱ | ✱ | ✱ | ✱ | ✱ | - | 7 |
| Ning Ding (2022) (36) | - | ✱ | ✱ | ✱ | ✱ | ✱ | ✱ | ✱ | 8 |

CarolinaDonat-Vargasa (2019) is case control study. In the case control study, 1. Is the case definition adequate? 2. Representativeness of the cases; 3. Selection of controls; 4. Definition of controls; 5. Comparability of cases and controls on the basis of the design or analysis; 6.Ascertainment of exposure; 7. Same method of ascertainment for cases and controls; 8. Non-Response rate. Pi-I D. Lin (2020), Andrea Winquist (2014) and Ning Ding (2022) are cohort studies. In the cohort studies, 1. Representativeness of the exposed cohort; 2. Selection of the non exposed cohort; 3. Ascertainment of exposure; 4. Demonstration that outcome of interest was not present at start of study; 5. Comparability of cohort on the basis of the design or analysis; 6. Assessment of outcome; 7. Was follow-up long enough for outcomes to occur; 8. Adequacy of follow up of cohort. “✱”：One point .

**Table S2.** Agency for Healthcare Research and Quality (AHRQ) quality assessment scale

| **First Author (year)** | **Quality Indication from AHRQ Scale** | | | | | | | | | | | **Scores** |
| --- | --- | --- | --- | --- | --- | --- | --- | --- | --- | --- | --- | --- |
|  | 1 | 2 | 3 | 4 | 5 | 6 | 7 | 8 | 9 | 10 | 11 |  |
| Jin-Young Min (2012) (26) | ✱ | - | - | ✱ | ✱ | ✱ | ✱ | ✱ | - | ✱ | - | 7 |
| Sarah Dee Geige (2014) (22) | ✱ | - | - | ✱ | - | ✱ | ✱ | ✱ | - | - | - | 5 |
| Krista Y. Christensen (2016) (29) | ✱ | - | - | ✱ | ✱ | ✱ | ✱ | ✱ | - | - | - | 6 |
| Wen-Wen Bao (2017) (31) | ✱ | - | - | ✱ | ✱ | ✱ | ✱ | ✱ | - | ✱ | - | 6 |
| Aimin Chen (2019) (35) | ✱ | - | - | ✱ | ✱ | ✱ | ✱ | ✱ | - | ✱ | - | 7 |
| Gisella Pitter (2020) (30) | ✱ | - | - | ✱ | - | ✱ | ✱ | ✱ | - | - | - | 5 |
| Shengen Liao (2020) (25) | ✱ | ✱ | - | ✱ | ✱ | ✱ | ✱ | ✱ | - | ✱ | - | 8 |
| Xin Mi (2020) (32) | ✱ | - | - | ✱ | ✱ | ✱ | ✱ | ✱ | - | ✱ | - | 7 |
| Maria Averina (2021) (23) | ✱ | - | - | ✱ | ✱ | ✱ | - | ✱ | - | ✱ | - | 6 |
| Maryam Zare Jeddi (2021) (28) | ✱ | ✱ | - | ✱ | - | ✱ | ✱ | ✱ | - | - | - | 6 |
| Shu Yu (2021) (34) | ✱ | - | - | ✱ | ✱ | ✱ | ✱ | ✱ | - | ✱ | - | 7 |

1. Define the source of information(survey,record,review); 2. List inclusion and exclusion criteria for exposure and unexposed subjects (cases and controls ) or refer to previous publication; 3. Indicate time period used for identifying patients; 4. Indicate whether or not subjects were consecutive if not population-based; 5. Indicate if evaluators of subjects components of study were masked to other aspects of the status of the participants; 6. Describe any assessment undertaken for quality assurance purpose(e.g., test/ retest of primary outcome measurements); 7. Explain any patient exclusions from analysis; 8. Describe how confounding was assessed and/ or controlled; 9. If applicable, explain how missing data were handle in the analysis; 10. Summarize patient response rates and completeness of data collection; 11. Clarify what follow-up, if any, was expected and the percentage of patients for which incomplete data or follow-up was obtained. “✱”：One point .

**Table S3** Criteria for the risk of bias assessment of each included study.

| **Bias** |  | **Risk of Bias Domains and Ratings** | **Answer** |
| --- | --- | --- | --- |
| **Key Criteria** | **Detection bias, exposure assessment** | Can we be confident in the exposure characterization? | -LOW risk: There is high confidence that the exposure to residential greenness is the true average population exposure.  -PROBABLY LOW: There is indirect evidence that suggests low risk of bias.  -PROBABLY HIGH risk: There is insufficient information to permit a judgment of high risk of bias, but there is indirect evidence that suggests high risk of bias.  -HIGH risk: There is direct evidence of high risk of misclassification bias. |
|  | **Detection bias, outcome assessment** | Can we be confident in the outcome assessment? | -LOW risk: Outcome was classified based on diagnosis standard criteria (International Classification System code) and provided by a national or regional database.  -PROBABLY LOW: Outcome was assessed based on diagnosis standard criteria and collected by researcher  -PROBABLY HIGH risk: Outcome was not assessed based on standard diagnosis criteria AND is accompanied by validation sub-study or sensitivity analysis to suggest that the risk is minimum.  -HIGH risk: Outcome was assessed based on self-reports (parents, family) and data collected by the researcher. |
|  | **Confounding bias** | Did the study design or analysis account for important confounding and modifying variables? | -LOW risk: Study accounted for all important confounders which were measured consistently  -PROBABLY LOW: Study accounted for most of confounders AND is not expected to introduce bias  -PROBABLY HIGH risk: Study accounted for some but not all of confounders AND is expected to introduce bias  -HIGH risk: Study did not account for potential confounders OR were inappropriately measured |
| **Other Criteria** | **Selection bias** | Did selection of study participants result in appropriate comparison groups? | -LOW risk: The descriptions of the studied population were sufficiently detailed to support the assertion that risk of selection effects was minimal.  -PROBABLY LOW risk: There is insufficient information about population selection to permit a judgment of low risk of bias, but there is indirect evidence that suggests low risk of bias.  -PROBABLY HIGH risk: There is insufficient information about population selection to permit a judgment of high risk of bias, but there is indirect evidence that suggests high risk of bias.  - HIGH risk: There were indications from descriptions of the studied population of high risk of bias. |
|  | **Attrition/exclusion bias** | Were outcome data complete without attrition or exclusion from analysis? | -LOW risk: There were no missing outcome data or missing data unrelated to true outcome  -PROBABLY LOW: There was insufficient information about incomplete data to judge for low risk, but indirect evidence that suggests low risk of bias  -PROBABLY HIGH risk: There was insufficient information about incomplete data to judge for high risk, but indirect evidence that suggests high risk  -HIGH risk: Missing outcome data is related to true outcome |
|  | **Selective reporting bias** | Were all measured outcomes reported? | -LOW risk: All of the studies pre-specified outcomes and findings are reported  -PROBABLY LOW: There was insufficient information about selective outcome to judge for low risk, but indirect evidence that suggests study was free of selective report  -PROBABLY HIGH risk: There was insufficient information about selective reporting to judge for high risk, but indirect evidence suggests that study was not free of selective reporting  -HIGH risk: Not all pre-specified outcomes and findings were reported, or one/more of the primary outcomes or analyses were assessed or executed with other methods than the pre-specified one, or one/more of the reported outcomes/findings was/were not pre-specified |
|  | **Conflict of interest** | Potential source of bias in reporting through source of funding | -LOW risk: The study did not receive funding from an entity with financial interest in the outcome of study  -PROBABLY LOW: There is insufficient information to judge for low risk, but indirect evidence suggests study was free of financial interest  -PROBABLY HIGH risk: There is insufficient information to judge for high risk, but indirect evidence suggests study was not free of financial interest  -HIGH risk: The study received support from an entity with financial interest in the outcome of study |
